# Advancing non-invasive melanoma diagnostics with deep learning and multispectral photoacoustic imaging

**DOI:** 10.1016/j.pacs.2025.100743

**Published:** 2025-06-19

**Authors:** Aboma Merdasa, Alice Fracchia, Magne Stridh, Jenny Hult, Emil Andersson, Patrik Edén, Victor Olariu, Malin Malmsjö

**Affiliations:** a Department of Clinical Sciences Lund, Ophthalmology, Lund University, Sweden; b Skåne University Hospital, Lund, Sweden; c Computational Science for Health and Environment, Centre for Environmental and Climate Sciences, Lund University, Sweden

**Keywords:** Melanoma, Clinical translation, Photoacoustic imaging, Deep learning, Spectroscopy

## Abstract

The incidence of melanoma is rising and will require more efficient diagnostic procedures to meet a growing demand. Excisional biopsy and histopathology is still the standard, which often requires multiple surgical incisions with increasing margins due inaccurate visual assessment of where the melanoma borders to healthy tissue. This challenge stems, in part, from the inability to reliably delineate the melanoma without visually inspecting chemically stained histopathological cross-sections. Spectroscopic imaging have shown promise to non-invasively characterize the molecular composition of tissue and thereby distinguish melanoma from healthy tissue based on spectral features. In this work we describe a computational framework applied to multispectral photoacoustic (PA) imaging data of melanoma in humans and demonstrate how the borders of the tumor can be automatically determined without human input. The framework combines K-means clustering, for an unbiased selection of training data, a one-dimensional convolutional neural network applied to PA spectra for classifying pixels as either healthy or diseased, and an active contour algorithm to finally delineate the melanoma in 3D. The work stands to impact clinical practice as it can provide both pre-surgical and perioperative guidance to ensure complete tumor removal with minimal surgical incisions.

## 1 Main

Cutaneous melanoma is the most lethal form of skin cancer, and accounts for about 55,500 deaths worldwide per year [1]. Early-stage melanoma is highly curable, whereas the management of metastatic melanoma is challenging. The standard diagnostic procedure requires excisional biopsy and histopathological analysis to assess histomorphological, topographical and immunohistochemical features [2]. Diagnostic biopsies face challenges including inaccurate histopathological tumor border determination, imprecise staging, and misdiagnosis, resulting in inadequate treatment [3], [4]. Larger margins increase the likelihood of a radical excision at the expense of removing excess of healthy tissue, which oftenonly requires cosmetic treatment. However, if the melanoma is in a sensitive region, removing too much tissue can affect body function, such as eye-lid tumors [5]. If a lesion is suspected of being melanoma, a diagnostic surgical biopsy is commonly first performed [6]. If the lesion is confirmed to be a melanoma, a second wider excision is performed to ensure complete removal of the melanoma [7]. The width of the second excision is determined by the Breslow’s depth, which is a measure of tumor depth in millimeters from the top layer of the epidermis (stratum granulosum) to the tumor's deepest point determined via histopathology. Breslow’s depth correlates with melanoma invasiveness and prognosis: the deeper the melanoma has grown into the tissue, the greater the risk of spread and the poorer the outcomes. Being able to pre-surgically determine the melanoma depth could therefore have tremendous impact on clinical practice.

To date, there are no clinically established techniques that can non-invasively determine the Breslow’s depth of a melanoma skin tumor. This technical gap limits the ability to inform on the optimal excision margins of melanomas. As a result, complete removal on the first attempt becomes challenging, leading to increased reoperations, patient suffering, and higher healthcare costs. Conventional clinical imaging (i.e. ultrasound, MRI, CT, PET) face various challenges, such as lack of endogenous molecular contrast, requiring molecular tracers, instrument complexity, or limited spatial resolution for the purposes of diagnosing skin tumors, which consequently requires histopathology for diagnosis to be made. Photoacoustic (PA) imaging is an emerging method that is capable of indirectly characterizing the optical properties of tissue using laser light to illuminate and high frequency ultrasound to detect. Absorption of photons in tissue causes the tissue to expand when the absorbed energy is quickly released as heat, which in turn causes a pressure wave that is measured with an ultrasound transducer. As such, PA imaging can spatially resolve both the structure and absorption properties of tissue at several centimeters depth [8]. This makes it a promising imaging modality for assessing the Breslow’s depth [9], [10]. Perhaps more intriguing is its potential to provide a complete 3D delineation of the melanoma skin tumor, which is of extreme value for both pre-surgical and perioperative guidance.

The need for unbiased and automated identification of image features has fueled the implementation of deep learning in clinical imaging [11]. Algorithms for skin tumor segmentation have been demonstrated employing convolutional neural networks (CNNs), pre-trained deep CNNs or residual neural networks. However, these algorithms have only been applied to dermatoscopic [12] and hyperspectral images [13] to characterize the superficial spread of melanomas. Traditionally, these networks employ a training set of images where the tumors are either delineated by hand, or automatically generated through some feature identifying pre-processing algorithm, in order to predict the tumor border in unlabeled images [14]. While this may seem promising, it has little value for clinical use since the skin tumor borders are not reliably drawn by hand. The obstacle lies in the fact that the spatial features that are prominent in the images result from the limited spectral information standard imaging equipment can relay, which is not sufficient for generating a visual contrast of where the melanoma border should be drawn. This is mainly the reason why dermatoscopy or visual inspection is not adequate for determining the superficial spread of the melanoma, where the necessary spectral contrast obtained through histochemical staining of a biopsy becomes necessary to generate the needed visual spectral contrast so that the borders of the tumor, and Breslow's depth, can be determined.

We recently suggested that spectral information, from hyperspectral imaging (HSI), can provide a viable alternative to circumvent the obstacle of having to rely on labelled images for training data [15]. Instead of using the spatial information in pre-labelled images, the spectral features from selected regions representing healthy tissue and tumor are used as the training data. Here, we extend the application of so-called *spectroscopy-driven* machine learning to photoacoustic imaging data of melanomas, which has not been done before. We present a fully automated computational framework that not only determines the Breslow’s depth, but also completely delineates the melanoma in 3D. What sets our approach apart is that the training data is generated uniquely for each patient based on the PA spectroscopic signatures of healthy tissue and tumor for each patient. This important feature circumvents the need for larger population-based training data to universally identify features for healthy tissue and melanomas, which both vary largely across the population. Moreover, the framework generates the training data without the need for human input via K-means clustering, removing any human bias in the diagnostic procedure. The application of spectroscopy-driven deep learning to photoacoustic imaging data is therefore promising and of high relevance for improving current clinical practice.

## 2 Results

### 2.1 Generating training data from spectral information

The first step toward a fully autonomous delineation procedure is to generate a training data set without human input for each sample. Here, we rely on the spectral features and the fact that melanoma tumors exhibit increasingly higher absorption at shorter wavelengths due to the higher melanin content. Thus, the spectral intensity information in each pixel is used by K-means clustering to separate all pixels into two initial clusters: healthy and non-healthy. To ensure a spatial separation between training points and avoid labelling areas of uncertainty, a margin of 0.5 mm between these clusters is left unlabeled. This area will effectively be classified by the 1-D CNN, defining the prediction map for tumor border.

**Fig. 1:**
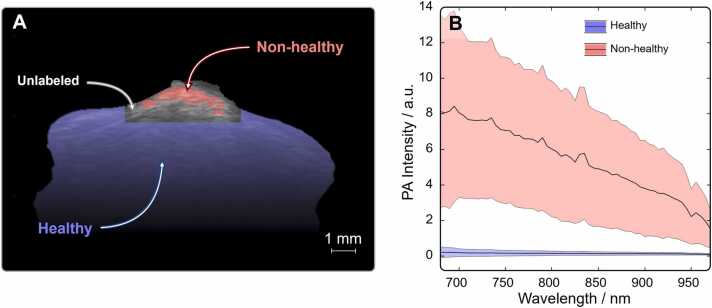
Generating training data from spectral information in a PA image. (A) Partial vertical cross-section of a PA image showing the tumor pixels clustered with K-means for generating training data for the model: in red, the high PA intensity pixels, corresponding to high melanin content therefore labeled as non-healthy points; in blue, the healthy part of the sample; in grey, remaining points that are left unlabeled close to the undefined tumor border. Note that the region of the unlabeled class has been extended with a minimal distance from non-healthy pixels to provide room for the model to predict unlabeled pixels. (B) Spectra taken as an average from the pixels of the two clusters representing non-healthy (red) and healthy (blue) pixels.

The results for a representative example in a single cross-section measurement are demonstrated in Fig. 1A, where each pixel receives a color coding only if it is either healthy (blue) or non-healthy (red), while unlabeled pixels remain grey. While information in space is utilized here, we emphasize that spatial information was not used to obtain the information contained in the clusters.

We note that the clustering is performed on all pixels in the entire 3D volumetric data set, after which pixels in each individual cross-section is labelled as shown in Fig. 1A. This approach, as opposed to applying the cluster analysis on each cross-section individually, is necessary to avoid identifying non-healthy pixels in every measured cross-section since the tumor is not expected to extend across the entire biopsy. The depicted image also shows a vastly larger amount of healthy pixels compared to non-healthy, and in order to avoid sampling bias where the model develops a preference toward the majority class, which consequently impacts the sensitivity and specificity of the minority class, we randomly select an equal number of training points from each class which are used as training data in the model (see Methods for details) [16], [17].

Fig. 1B shows the spectra calculated as an average from all pixels represented by each cluster labelled as either healthy or non-healthy tissue. The spectral difference in terms of intensity across the entire spectral range becomes evident for the two clusters. Moreover, the large variation for the non-healthy spectra is expected due to a gradual increase in melanin depending on the region of the tumor a pixel-spectrum is extracted from. Moreover, the higher variation toward shorter wavelengths is simply due to the absorption coefficient of melanin being higher, which results in a larger variance. At this stage we have managed to produce the training data without manually specifying any spatial or spectral feature. The only parameter we have set is the number of clusters, which will remain the same (2) for all analyzed skin tumors.

K-means clustering has previously been used in the process to delineate skin tumor borders in dermatoscopy images, however, this was for the purpose of first removing spatially varying features (i.e. hair, ink) and subsequently generate training data to be used in a prediction model, which is still based on spatial features [18]. In our framework, the clusters are used to generate spectra which the prediction model then use to classify pixels, which consequently are more reliably characterized since the information contained in the spectra are tied to the molecular composition of the tissue. Our approach is more feasibly implemented clinically for non-invasively assessing Breslow’s depth with deep learning methods since pre-labelled images delineating the melanoma at a depth for training a model can currently not be produced without histopathology.

### 2.2 Tumor depth prediction with CNN

**Fig. 2:**
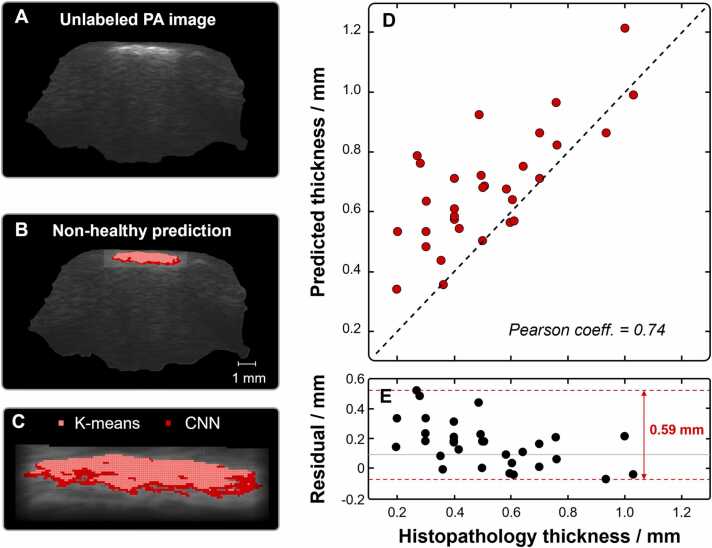
Results from applying CNN. (A) Unlabeled PA image. (B) Pixels predicted to represent non-healthy samples marked in red color. (**C**) Close-up of the predicted non-healthy region comparing results from K-means (light red) and 1D-CNN (dark red). (**D**) Correlation plot comparing the histopathological thickness and predicted thickness where a Pearson correlation coefficient of 0.74 is obtained. (**E**) The residual data from each predicted thickness in relation to the reference (histopathology) where an error spanning approximately 0.6 mm is obtained, with a predominant overestimation of the melanoma thickness. Note that the x-axis is shared between panel **D** and **E**.

The tumor thickness (Breslow’s depth) was predicted in the PA images by first acquiring spectra representing the two training data clusters, which were subsequently used to train a one-dimensional convolutional neural network (1D-CNN) model. The 1-D CNN model performed convolution across the spectral range, taking only one pixel-spectrum as an input at a time. Using the trained model, we then classified each pixel-spectrum belonging to the unlabeled cluster to either belong to the healthy or non-healthy cluster with a probability ranging between 0 and 1, respectively. A prediction of 0–0.5 is classified as healthy and above 0.5 is classified as non-healthy. Fig. 2A-C demonstrate the pixel-based prediction results superimposed on top of a gray-scale PA image where a comparison between the clustering of non-healthy pixels from K-means (light red) and the pixel prediction from 1D-CNN model (dark red) is shown. The pixel-based prediction was performed on every cross-section in a single sample. We thereafter automatically determined which cross-section contained the most non-healthy pixels extending vertically into the sample to obtain the maximum tumor thickness, which is the value that is compared to the histopathological diagnosis.

A key feature of our approach is that each model instance is trained using data from a single patient only. By handling each patient individually, we avoid introducing any patient-to-patient biases and circumvent the problem of distributional shift in the data. Thus, one framework using the same set of parameters for all samples, generates a unique training data set for each patient. We used our model to calculate tumor thickness for all 31 samples and compared the results to the ground truth tumor thickness determined by conventional histopathology. Notably, when we state that our model was applied to multiple samples, it means that a separate but identical model instance was trained individually for each sample, effectively having different training data. Fig. 2D shows the correlation plot where a Pearson correlation coefficient of 0.74 is calculated, suggesting a good correlation of our method compared to histopathology. The results indicate that the 1D-CNN applied to PA data slightly overestimates the tumor depth, which from a clinical perspective is the preferred direction to err in to ensure radical excision. Possible reasons for this systematic offset are discussed further below. Fig. 2E illustrates the residuals for each predicted tumor thickness compared to the reference values obtained from histopathology, revealing an error margin of approximately 0.6 mm, with a tendency to overestimate melanoma thickness.

### 2.3 Automatic skin tumor delineation

The Breslow’s depth is an important diagnostic measure both for the purpose of determining excision margins as well as assessing the risk of metastasis, for which the 1-D CNN model prediction will suffice. The first step toward providing surgical guidance for radical excision is to visualize the tumor's 3D architecture and accurately delineate its borders. Moreover, the CNN predictions are affected by noise from hair, surgical markers, sutures, and incision artifacts, emphasizing the need for better segmentation to minimize noise and accurately define tumor boundaries.

**Fig. 3:**
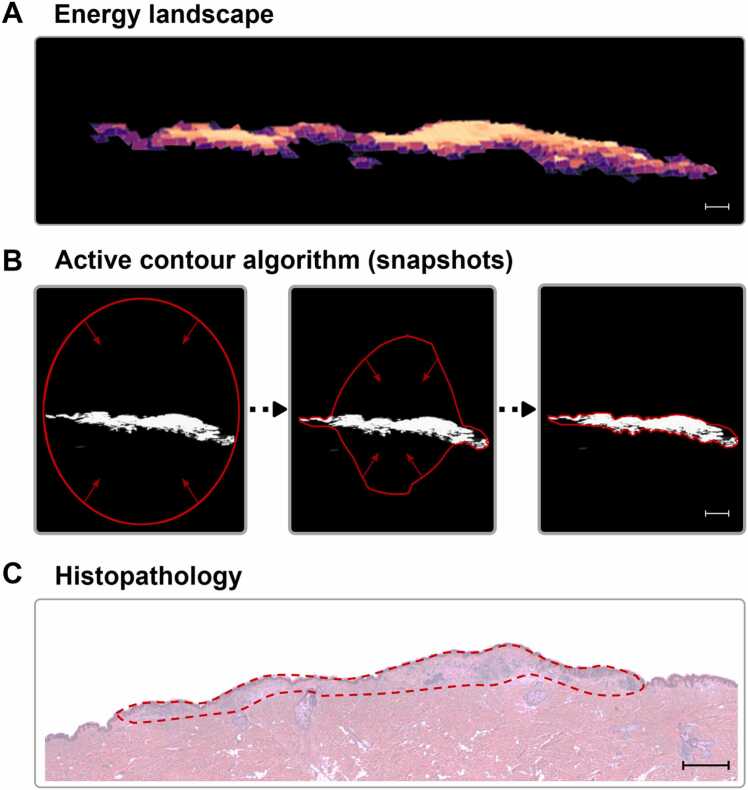
Delineating the melanoma with an active contour algorithm. (A) Energy landscape of a vertical cross-section of a representative PA image of melanoma. (B) Snapshots of the active contour progression showning the initial (left), intermediate (middle), and final (right) positions. (C) Corresponding histopathology cross-section showing a similar shape identified as tumor by the pathologist. The scale bar in all images represents 1 mm.

To include the spatial information in the data, we propose a customized active contour segmentation algorithm, adapted from recent study by some of us [15], specifically for PA imaging data to effectively delineate irregularly shaped objects. Initially, the CNN-predicted tumor volumes are used as input from which an energy landscape that spatially contrasts healthy and non-healthy regions is generated. This is achieved by applying a concept that can be likened to the pouring of sand in a single point, from which the sand spreads laterally as the amount increases. A higher CNN-generated probability that a pixel-spectrum represents a tumor, the more “sand” will be poured on that pixel. Pixel-spectra classified as healthy act as finite sinks. Setting a limit that sand is only shared with directly neighboring pixels, an energy landscape that removes small regions of incorrectly classified pixel-spectra can be obtained (Fig. 3A). The contour algorithm then minimizes an energy cost function shaped by internal contour properties (e.g., stiffness, homogeneity, gravitational pull) and external image properties (e.g., intensity), reducing prediction noise and artifacts (see Methods for details). Fig. 3B shows a few snapshots of the active contour algorithm acting on the energy landscape to finally settle on a region that ultimately is used as the final delineation of the melanoma tumor (see video S1 for complete process). Fig. 3C shows the histopathological cross-section from the same sample and approximate location where similarities in both shape and dimensions can be seen. This demonstrates the capability of our approach to non-invasively and automatically delineate skin tumor borders and generate images of the tumor architecture that closely resemble histopathological results.

Supplementary material related to this article can be found online at doi:10.1016/j.pacs.2025.100743.

Video S1

### 2.4 3D visualization of the tumor with PA imaging for pre-surgical guidance

For effective surgical guidance to ensure complete tumor removal, the goal is to provide a pre-surgical visualization of the entire skin tumor in relation to healthy tissue with a clear outline of its borders. As previously described, the PA measurements were performed in consecutive cross-sections separated by approximately 0.5 mm. Thus, the spatial resolution in the scan direction is much lower than the in-plane resolution (∼ 0.1 mm). To account for this, we apply *Frame Interpolation for Large Motion (FILM)* [19] to interpolate between cross-sections and generate data for a more continuous and smooth final 3D representation. The interpolation algorithm is AI-based and aims to find the most natural transition between two images according to similarities of spatial features. The user defines the number of steps for this transition, which we calibrated to obtain a resolution comparable to the in-plane spatial resolution. We note that this user input is purely for visualization purposes and is not an input required for any classification.

**Fig. 4:**
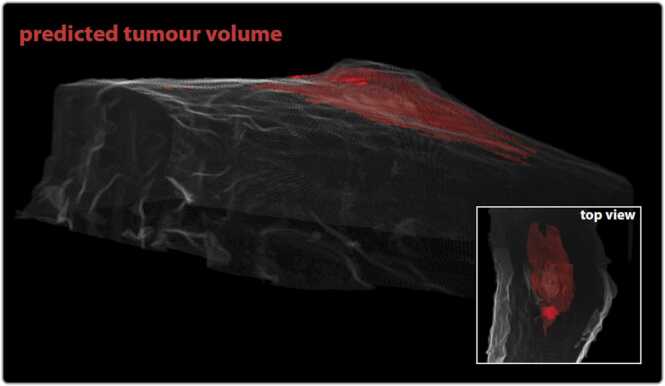
Visualizing the melanoma in 3D. Representative visualization of predicted tumor volume (red) in relation to surrounding healthy tissue (semi-transparent gray). This 3D reconstruction is based on PA measurements taken in consecutive cross-sections approximately 0.5 mm apart to which the FILM Google Network has been applied to match the spatial resolution in all three dimensions. The scale bar in the image represents.

To enhance computational efficiency, interpolation is applied only to the contours derived from the measured cross-sectional predictions. This approach avoids conducting the full analysis, including generating prediction maps, on the interpolated images. It is worth noting that the interpolation in practice operates on spatial features obtained via prediction maps that are generated from contrasting spectral features and is therefore tied to the molecular composition of the sample. Fig. 4 shows a representative example of a tumor that is visualized from a couple of different directions in red against the backdrop of healthy tissue (semi-transparent).

## 3 Discussion

### 3.1 Previous PA imaging of melanoma in patients

Beyond several animal studies using PA imaging for characterization of melanoma tumors [20], [21], [22], only a few studies report on the non-invasive determination of melanoma tumor borders using PA imaging on humans where either a few or several excitation wavelengths are used. In a previous study, we examined 52 suspected melanomas *ex vivo* using PA imaging with 59 excitation wavelengths where we demonstrated the ability to assess the tumor depth non-invasively [10]. However, like some other studies [9], [23], [24], [25], spectral unmixing was used requiring user input, rendering the approach non-autonomous and less adaptable to the expected spectral variability between patients. To the best of our knowledge, this is the first study implementing a deep learning algorithm that autonomously adapts to the expected inter-individual signal variability between samples and determines the skin tumor borders in 3D without any user input. Moreover, the current computational framework retrains the model on each instance (patient), which is new. Our approach also does not require any labeling of spatial features, which makes it more realistic for clinical implementation.

### 3.2 Deep learning for skin tumor delineation

A recent review by Mirikharaji et al [14]. provides a comprehensive overview of the development of deep learning for superficial skin tumor segmentation [26]. Here, it becomes evident that the data sets available include only images acquired via regular RGB cameras or via dermatoscopy. Two primary problems exist with the available data sets, which is the lack of manually segmented data for training, and the overall insufficient statistical representation of all different sub-types. From the perspective of the traditional application of deep learning algorithms on segmented images trained on spatial features, this is indeed a problem. We recently reported on the necessary transition from identifying spatial features to spectral features to circumvent the challenge of requiring a segmented tumor for training [15]. This approach becomes even more relevant when transitioning to delineating the tumor within the tissue, as demonstrated in this work. Beyond bypassing the need for labelled training images, our approach trains on the spectroscopic features which has greater potential to replicate the information obtained through histopathology. The spectroscopy-driven deep learning approach thus has higher clinical relevance than the conventional method of using labelled spatial features.

### 3.3 Toward precision skin tumor diagnostics

Current work relies on the characterization of the optical properties of tissue in which healthy tissue is contrasted against melanoma, making the approach individualized by nature. The algorithm itself does not adapt to the individual sample, but the training data and prediction model it produces become unique, which consequently leads to an individualized model tied to the patient. Moreover, it is worth noting that the approach is also unique to each instance, although the spectral features will be more similar with each instance within the same patient. This feature is of high relevance to the emerging field of precision diagnostics [27]. While the spectral information is closely tied to the molecular composition, we do not provide a characterization of the complete molecular profile of the individual. However, the relative changes are still partially unique to the individual which is also accounted for by the model. Thus, our approach actively moves away from attempting to identify a universal fingerprint for melanoma skin tumors, as well as healthy tissue, which may be futile due to the expected interindividual variability even in healthy tissue [28].

### 3.4 Employing information in multiple measurement domains

Throughout this work we have emphasized the importance of spectroscopy in the generation of the prediction map. However, the described approach also relies on the spatial information in the sense that neighboring pixels slightly influence the final delineation. While in most cases, this step does not change the result significantly, one can consider cases where features such as hair follicles, that naturally contain melanocytes, could initially be classified as non-healthy pixels while an active contour algorithm with optimized parameters can exclude such unwanted objects in the end result. This optimization certainly requires additional measurements where such objects can be identified and properly accounted for. In current work, we also take advantage of the generally higher PA intensity produced by a strong absorption of higher energy photons in the melanoma to generate the training data via K-means clustering. While a higher intensity at a particular wavelength is simply a smaller part of a spectral feature, our results suggest that multispectral PAI with just a few excitation wavelengths could still be used to automatically generate training data for a prediction model. Thereby, we exploit several measurement domains produced in a single multispectral PA acquisition where each contributes to a specific step in the automated process for the generation of: training data (intensity), prediction map (spectra) and tumor delineation (spatial).

### 3.5 Wavelength selection optimization

Data across the entire spectral range (690–970 nm) was used in the analysis where no optimization was made for identifying the minimal number of relevant wavelengths that can be used without sacrificing prediction performance. Including all wavelengths mainly serves a purpose in this exploratory phase since the model blindly looks for changes in spectral features rather than specifically searching for spectral features related to known tissue or tumor chromophores. With sufficient data, ensuring inter-individual variations and different melanoma tumor sub-types can be accounted for, an optimization algorithm can be applied. Such an approach could hypothetically include intensity from different combinations of excitation wavelengths to assess model performance. For clinical implementation feasibility, the number of excitation wavelengths should be minimized in order to expedite the measurement and thus reduce potential artifacts caused by patient movement.

### 3.6 Correlation offset

The results comparing Breslow’s depth determined with our approach to that of conventional histopathology show that our model systematically overestimates the melanoma thickness by 0.6 mm at most for all 31 measured samples. The histological margin needs to be at least 2 mm to be considered radical and avoid the need for further surgery with extended excision [29]. Therefore, our margin of error falls well within the clinically accepted range for safe tumor removal, although in practice a margin in addition to the borders we determine should still be considered. Furthermore, our technique results predominantly in an overestimation, which is certainly safer than the potentially severe consequences of underestimating the tumor depth in a diagnostic setting. We note that the samples are placed in formalin right after being measured with PAI before being sent for histopathology, which reportedly causes the entire sample to shrink [30]. This could certainly be a reason for why there is a systematic offset and should be further studied to understand the extent of it. There are also no reports on the impact of staining on the physical size of the sample and to what extent it could contribute to a systematic offset, which also needs to be understood for further improving accuracy in the non-invasive melanoma thickness assessment.

### 3.7 Clinical impact

The current study provides an approach to non-invasively visualize melanoma tumors in 3D. The tumor dimensions obtained with the described approach are in good agreement with histopathology. Since histopathological cross-sections are around 5 µm thick and only a few locations across the 2–5 cm long biopsy are examined, a 3D reconstruction of the melanoma architecture can provide a foundation to yield more information compared to histopathology. However, we emphasize that the intention is not to replace histopathology, but rather to enable pre-surgical (before surgery) measurement of tumor depth to facilitate radical excision on the first surgical attempt. As an intermediate step to pre-surgical guidance, a 3D reconstruction could guide pathologists in terms of where along the sample a cross-section should be made to reduce the risk of missing a non-radical excision.

Current clinical praxis involves first performing a diagnostic excision of a suspected melanoma with a 2 mm margin. If the suspicion is confirmed, a second, wide local excision is performed around the site, with a margin width determined by the histopathologically assessed tumor thickness. European guidelines recommend a wide local excision with a 1 cm margin for melanomas that are ≤ 2.0 mm thick, while a 2 cm margin is recommended for thicker melanomas [29]. A sentinel lymph node biopsy is recommended for primary melanomas thicker than 1.0 mm [31]. If a non-invasive assessment of the melanoma thickness could be performed pre-surgically, surgeons could optimize the margins of the initial excision, avoiding multiple procedures and achieving complete tumor removal on the first attempt.

Another interesting clinical application is the potential for rapid peri-operative (during surgery) diagnostics. The described technique provides an automated tumor outline after rapid scanning of the excised tumor, allowing the surgeon to receive feedback on whether the tumor has been excised radically within minutes. Considering the current un-optimized algorithm takes a few minutes to generate a 3D prediction map of a melanoma tumor, it is of high relevance to be applied in the context of Mohs micrographic surgery, where the patient remains in surgery while the excised biopsy is examined with an expedited form of histopathology that currently has a turnaround time of a few hours.

## 4 Summary

This work for the first time applies machine learning for automatic delineation of melanomas in 3D, which was enabled by photoacoustic imaging data. The non-invasively determined melanoma thickness show a strong correlation with measurements obtained through histopathological examination with an error well within clinical acceptance criteria. Moreover, we demonstrate how a complete delineation of the melanoma in 3D can be obtained non-invasively for both pre-surgical and peri-operative guidance. One algorithm generates a unique set of training data for each patient, which actively moves away from the conventional deep learning tumor segmentation approach of establishing a universal signal feature for both healthy tissue and melanoma. This bypasses the need for vast amount of data to generate a reliable prediction model, which is challenging considering the expected large inter-individual variation of patient-related features. The spectroscopy-driven deep learning approach also circumvents the need for labeled images, which is currently not possible to generate non-invasively for tumor thickness assessment without histopathology. The described approach aims to open new pathways for the clinical translation of photoacoustic imaging integrated with machine learning for the application to personalized skin tumor diagnostics and other applications where the non-invasive assessment of the tissue molecular composition would be of value.

## 5 Methods

### 5.1 Patient characteristics and histopathological analysis

Patient characteristics were pseudo-anonymized and are summarized in Table 1. Suspected tumors were surgically removed under local anesthesia at the Department of Dermatology at Skåne University Hospital in Lund. Thereafter, they were transported to the Department of Ophthalmology where the PA measurements were performed. Within one hour, samples were placed in formalin and sent for histopathology. The excised biopsies were first divided into larger sections from which thinner slices were cut from the ends. These slices were then stained with hematoxylin and eosin and scanned where each cross-section was manually examined using Sectra IDS7 (Sectra AB, Linköping, Sweden). The dimensions of the lesion were determined by an experienced pathologist. Like the PA measurements, the cross-sections for histopathology were made perpendicular to the long axis of the biopsy. The PA measurements of the melanoma was then compared to those from the histological analysis. In all lesions the greatest PA tumor width was used for comparison.

**Table 1:** Patient characteristic with age, sex, Fitzpatrick skin type, assessed tumor depth and diagnosis (MM stands for malignant melanoma).

Age (years)	Sex (M/F)	Fitzpatrick type (I-IV)	Breslow depth (mm)	Diagnosis
58	F	II	0.36	Melanoma in situ
52	F	I	0.60	MM
38	M	II	0.50	MM in situ
53	M	II	0.90	MM
75	M	II	1.0	MM
69	M	II	0.75	MM
80	F	II	0.50	Lentigo melanoma
76	M	II	0.60	Lentigo melanoma
87	F	II	0.75	MM
51	M	I	1.0	MM
94	F	II	0.60	MM
48	M	I	0.60	Melanoma in situ
64	M	II	0.40	MM
61	F	II	0.27	MM in situ
69	M	II	0.50	MM in situ/little invasive component
44	F	II	0.30	MM in situ
53	M	II	0.65	MM in situ
87	M	II	0.50	MM
73	F	II	0.35	MM in situ
70	F	II	0.20	MM
77	F	I	0.50	MM in situ
79	M	II	0.40	MM
73	M	II	0.30	MM in situ
54	M	II	0.20	MM in situ
57	M	II	0.30	MM
48	M	II	0.28	MM in situ
70	M	II	0.40	MM in situ
71	F	II	0.40	MM in situ
69	M	II	0.40	MM in situ
71	F	I	0.70	MM in situ
76	F	II	0.70	MM

### 5.2 Photoacoustic imaging & data acquisition

Photoacoustic (PA) imaging is an emerging method for clinical use where non-invasive imaging of soft tissue is desired. It is promising from the fact that it can provide images reflecting the molecular composition of tissue at depths reaching several centimeters. A pulsed laser is typically used to populate the tissue with photons in a short period of time. A fraction of these photons is absorbed by the different tissue chromophores, after which a substantial portion of the absorbed photon energy is released as heat, which gives rise to a brief expansion and contraction of the tissue surrounding the absorbed chromophore. This so-called *thermoelastic expansion* produces a mechanical (acoustic) pressure wave that propagates back toward the surface of the tissue where it is detected by the high-frequency ultrasound transducer. Furthermore, by changing the excitation wavelength, the wavelength-specific absorption of chromophores allows them to be differentiated by absorption dependendent intensity of the acoustic signal. Hence, by the mechanism of exciting the tissue with light at several wavelengths and detecting its absorption indirectly via sound, allows for deep-tissue imaging of different chromophores.

In our case, PA imaging was performed using a Vevo LAZR-X multimodal imaging system (FUJIFILM VisualSonics Inc., Toronto, ON, Canada). The system employs 59 excitation wavelengths over a spectral range between 680 and 970 nm in 5 nm incremental steps. The repetition rate is 20 Hz with each pulse having a temporal width of 7 ns. The pulse fluence did not exceed 55 mJ/pulse, maintaining a safe photon dose for use on humans. For detection, a high-frequency linear ultrasound transducer was used with a central frequency at 40 MHz, resulting in an axial resolution of 50 µm and a later resolution of 110 µm. A bifurcated liquid light-guide was used to align the excitation laser parallel to the linear ultrasound transducer on both sides, and the height of both detection and excitation was optimized to ensure a uniform illumination and desired detection depth of the sample. Both fibers and the ultrasound transducer were attached to a stepper motor that scanned the acquisition system across the sample in incremental steps of 0.5 mm.

**Fig. 5:**
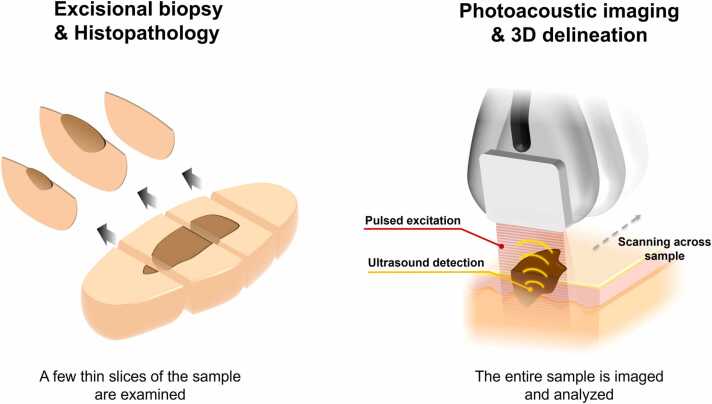
Schematic comparing histopathology and PA imaging. (**left**) In histopathology, the sample is divided into a few large sections from which thin slices are shaved on the ends. Each slice is then stained and examined manually by a pathologist. Only a few sections per sample are examined to ensure the excision is radical and to determine the Breslow’s depth. **(right)** The photoacoustic imaging acquisition system with the laser excitation guiding pulsed laser excitation to the surface of the sample into the tissue. The acoustic echo response from the thermoelastic expansion is detected with a high-frequency ultrasound transducer. The acquisition system is then scanned across the sample in steps of 0.5 mm until the entire sample is measured.

Two Prolene 6–0 sutures were sewn to each end of the lesion, and it was then mounted in a 100 × 70 x 50 mm Perspex container, filled with buffered saline solution. The bottom of the container was covered with an ultrasound-attenuating material. Fig. 5 schematically shows the procedure of how a biopsy is examined with histopathology and photoacoustic imaging. The comparison highlights the low sampling rate of histopathology, which only examines a few thin cross-sections (roughly 5 µm thick), compared to PA imaging where the entire sample is measured and analyzed.

We note that the excitation volume induced by the diffusely scattered photons in the tissue looks quite different than that of the effective detection volume of the linear ultrasound transducer, leading to an imperfect overlap of the two. This translates into the acquired 2D cross-sectional images representing different in-plane thicknesses with a non-linear dependence on depth. Moreover, the photon flux reduces as a function of the optical properties of the tissue layers, leading to a complex dependence of the detected signal on the absorbing tissue layers. Methods exist to account for this, which we have recently evaluated [32], however, further considerations in how the image should be constructed from the data need to be made, as explained further below.

### 5.3 Data acquisition and structure

Photoacoustic imaging measurements were performed shortly after suspected melanomas were excised, after which the biopsies were placed in formalin and sent for histopathological analysis. 31 melanomas were measured to produce volumetric data of an entire biopsy according to an acquisition procedure described above. The orientation of PA image cross-sections was aligned to match the orientation of the histopathological slice to better compare the results. The resulting data matrix for each sample is therefore 4-dimensional where each 3-dimenstional (3D) voxel (having three spatial coordinates) contains a PA absorption spectrum with spectral 59 elements. It is worth noting that the model is applied on pixel-spectra in each vertical PA image cross-section where the spatial information of each pixel is retained for collectively analyzing the prediction map to obtain spatial dimensions of the melanoma and to visualize it in 3D.

### 5.4 Pre-processing: background segmentation

The structural information contained in the ultrasound (US) data was used to create a mask to segment out most of the background noise by developing an automatic intensity thresholding algorithm. The sample's outline was then computationally separated from any additional noise, refined through smoothing algorithms and applied to the PA data. The US data thus provided a foundation to identify the borders of the entire sample and separate it from the background signal produced by the saline solution, as well as other artifacts tied to the acquisition system or procedure (i.e. bubbles in the US gel, protective plastic on the transducer etc.).

PA spectra representing the absorption for the full sample across the 59 excitation wavelengths were assessed in all pixels. Each pixel was characterized by a spectrum whose intensity and profile depend on the concentration of the light-absorbing chromophores in that point. Melanin absorption dominates the composition of melanomas, which reflect a high PA absorption toward shorter wavelengths in our acquisition range. Other components, such as hemoglobin, collagen, lipids and water content, might also vary in non-healthy tissues, contributing to a wide range of tumor spectra variations.

### 5.5 Pre-processing: Intensity information and data labelling

A common limitation of neural network training is the need for an extensive amount of diverse training data to cover all data variations. Moreover, without a definitive ground truth for the full sample, labelling can be challenging. We therefore implement a method which proposes personalized training by focusing on the relative spectral difference between tissue types for the singular individual. Under the assumption that the signal coming from the melanoma would yield a higher absorption due to the higher melanin content, a generally higher PA signal is more likely representative of tumorous rather than healthy tissue.

### 5.6 Applying the model: Training data generation with K-means clustering

By harnessing the large contrast in absolute intensity of the photoacoustic signal, we automatically generate training and validation datasets of spectra for each sample via K-means clustering. For each sample, the spectra of all pixels are clustered to systematically isolate the points with the highest intensity. The higher intensity cluster is labelled as tumorous, whereas the other cluster is chosen to represent healthy tissue. A boundary of 0.5 mm of depth between the two classes of points is left unlabeled due to the absence of ground truth for the real outline of the tumor.

### 5.7 Applying the model: 1-dimensional convolutional neural network details

**Fig. 6:**
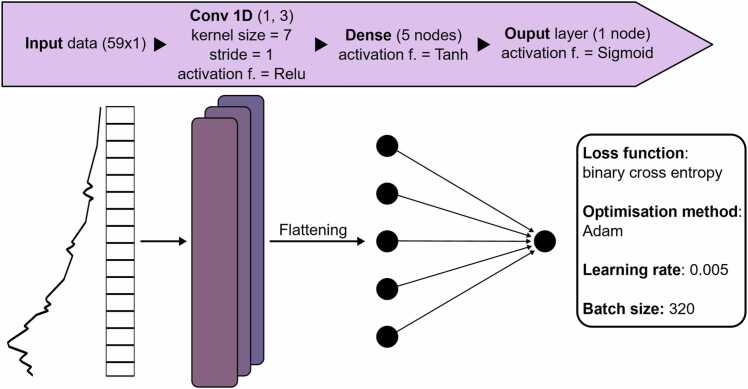
CNN hyperparameters and architecture.

A 1-dimensional CNN is chosen to classify each pixel according to their photoacoustic spectra. This method allows us to retain the information from all imaging wavelengths by analyzing the features of the spectral profile. The network architecture was chosen to be as simple as possible while still performing accurately. The kernel size was set to be sufficiently large to not identify spectral noise as features, but also to not average out potentially important features. We note that in the case for melanomas, the only expected distinguishing feature is a higher absorption toward shorter wavelengths, which in essence manifests as a different slope. However, the intention is for the model to exhibit versatility and be able to detect unexpected features as well. Details on the choice of model hyperparameters and optimization can be found below in Fig. 6. The model output represents the probability of a pixel belonging to each of the classes. Once the prediction map is obtained, the thickness of the predicted tumor region is automatically extracted for each slice.

### 5.8 Post-processing: active contour and 3-dimensional tumor visualization

The pixel-by-pixel output of our model can be further refined by including additional spatial information regarding the class identity of neighboring pixels. This can be done through an adaptation of the *sandpiles algorithm*, developed by Andersson et al. [12], which is customized to better perform on the data used herein. The algorithm builds an energy landscape for the final segmentation based on the prediction map generated by the 1-dimensional CNN. The energy landscape originating from the *sandpiles algorithm* serves as a guide to an active contour to segment the final tumor region[13]. This is classically implemented to depend on forces such as the gravitational pull from its center of mass, the contour stiffness and a homogeneous distribution of the contour points. The active contour algorithm was implemented according to the procedure outlined in [12] and adapted to better perform on our data with the parameters indicated in Fig. 6.

**Fig. 7:**
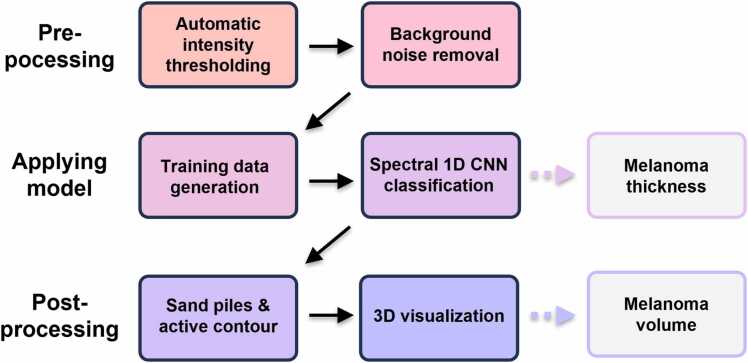
Schematic summarizing the data analysis procedure. The procedure is divided into three major steps where data is first pre-processed after acquisition to prepare the spectra for applying the model which yields the melanoma thickness. The post-processing here involves tuning the prediction map accounting for spatial features to visualize the tumor in 3D.

Due to the low resolution in the scan direction (0.5 mm) compared to the in-plane axial and lateral resolutions, a Google network (FILM) was used to interpolate between the experimentally acquired slices to balance the resolution along the longitudinal dimension of the sample. The interpolation is performed on the analyzed images where the tumor borders are already defined to reduce the computational load. With the information obtained from the interpolation, a final 3-dimensional outline of the predicted tumor region can be visualized and observed from essentially any direction. The entire data analysis procedure is summarized in Fig. 7, emphasizing the different steps involving the acquisition of the diagnostically important output parameters characterizing the melanoma thickness and its volume.

The dimensions of the interpolation have not been validated, and considering the issues of imperfectly overlapping excitation and detection volumes, further studies are needed to understand the uncertainty involved in digitally reconstructing the sample in the manner explained in this work. One can certainly argue that ensuring PA measurements are taken in the direction such that histopathological cross-sections align well with the measurements minimizes uncertainties related to interpolation. However, in order to advance the method for its intended use, which is to observe a 3D-constructed digital twin of the sample and tumor prior to excision, further measurements are needed to validate the interpolation.

## CRediT authorship contribution statement

**Aboma Merdasa:** Writing – review & editing, Writing – original draft, Visualization, Supervision, Project administration, Methodology, Investigation, Funding acquisition, Formal analysis, Data curation, Conceptualization. **Alice Fracchia:** Writing – review & editing, Writing – original draft, Visualization, Methodology, Investigation, Formal analysis. **Magne Stridh:** Writing – review & editing, Validation, Investigation, Data curation. **Jenny Hult:** Writing – review & editing, Validation, Supervision, Data curation. **Emil Andersson:** Writing – review & editing, Formal analysis, Data curation. **Patrik Edén:** Writing – review & editing, Supervision, Methodology, Conceptualization. **Victor Olariu:** Writing – review & editing, Writing – original draft, Supervision, Project administration, Methodology, Funding acquisition, Formal analysis, Conceptualization. **Malin Malmsjö:** Writing – review & editing, Writing – original draft, Supervision, Project administration, Funding acquisition, Conceptualization.

## Ethics

The protocol for this study was approved by the Ethics Committee at Lund University, Sweden (dnr). The research was performed according to the Declaration of Helsinki of 2008. Verbal and written information about the study and its voluntary nature was given to the patients, and written consent was obtained from all participants.

## Declaration of Competing Interest

The authors declare that they have no known competing financial interests or personal relationships that could have appeared to influence the work reported in this paper.

## Data Availability

Data will be made available on request.
